# Umbrellas Dangerous

**Published:** 1869-03

**Authors:** 


### UMBRELLAS DANGEROUS.

A GENTLEMAN has had his eye punched, and the sight irretrievably destroyed, by his walking up against the point of an umbrella, which a person in front of him was carrying under his arm or over his shoulder, and stopping suddenly on the street to speak to a friend; this ugly and inconsiderate habit of carrying umbrellas on the street, when the rain is not falling, is practised by a great many, who only need to have it mentioned to cause its rectification.

